# Neural Sensitivity to Social and Monetary Reward in Depression: Clarifying General and Domain-Specific Deficits

**DOI:** 10.3389/fnbeh.2019.00199

**Published:** 2019-10-09

**Authors:** Belel Ait Oumeziane, Olivia Jones, Dan Foti

**Affiliations:** Department of Psychological Sciences, Purdue University, West Lafayette, IN, United States

**Keywords:** social reward, monetary reward, depression, reward processing, event-related potentials

## Abstract

Reward dysfunction is thought to be play a critical role in the pathogenesis of depression. Multiple studies have linked depression to abnormal neural sensitivity to monetary rewards, but it remains unclear whether this reward dysfunction is generalizable to other rewards types. The current study begins to address this gap by assessing abnormal sensitivity to both monetary and social rewards in relation to depressive symptoms. We recorded event-related potentials (ERPs) during two incentive delay tasks, one with monetary reward and one with social reward. Both tasks were administered within the same sample, enabling a direct comparison of reward types. ERPs elicited by social and nonsocial rewards were morphologically similar across several stages of processing: cue salience, outcome anticipation, early outcome evaluation, outcome salience. Moderation analyses showed depression was linked with a pattern of general deficits across social and monetary rewards, specifically for the stages of outcome anticipation (stimulus-preceding negativity) and outcome salience (feedback-P3); self-reported reward sensitivity was generally associated with early outcome evaluation (reward positivity). Regression analyses modeling task-specific variance, however, showed a unique association between depression and outcome salience for social rewards, controlling for monetary rewards. The findings from this study underscore the importance of assessing neural sensitivity to multiple reward types in depression, particularly social reward. Characterizing the profile of reward functioning in depression across reward types may help to link laboratory-based deficits to relatively global vs. focal difficulties in real-world functioning.

## Introduction

Major depressive disorder (MDD) ranks among the most prevalent and economically onerous medical conditions, having an estimated lifetime prevalence rate of 16% (Kessler et al., 2003) and an annual cost of more than $80 billion (Greenberg et al., 2015). Given these alarming statistics, there has been a growing focus on better understanding the core pathophysiological processes of depression. A cardinal symptom is anhedonia; a lack of motivation and enjoyment of activities that are pleasurable (American Psychiatric Association, 2013). There has been a growing interest in translating findings from basic cognitive and affective neuroscience research to characterize anhedonia in depression in terms of quantitative deficits in reward functioning (Nestler and Carlezon, 2006; Pizzagalli et al., 2011; Russo and Nestler, 2013). In the current study, we focus on reward processing in the context of social and nonsocial domains across various stages of processing to better characterize the nature of the impairments in depression.

There has been converging evidence of reward dysfunction in depression across multiple units of analysis, including behavioral, neuroimaging, and electrophysiological research. Existing behavioral studies have linked depression with a rigid response style that is insensitive to reward contingencies (Henriques and Davidson, 2000; Pizzagalli et al., 2008), which is linked to anhedonia severity (Pizzagalli et al., 2005) and prospectively predicts poor treatment outcome (Vrieze et al., 2013). Building upon this behavioral data, functional magnetic resonance imaging (fMRI) studies have shed light on the pathophysiology of reward functioning in depression. For example, studies found decreased reactivity to rewards in the striatum, including the caudate, putamen, and nucleus accumbens (Steele et al., 2007; Knutson et al., 2008; Forbes et al., 2009; Pizzagalli et al., 2009; Moses-Kolko et al., 2011). These regions comprise the mesocorticolimbic dopamine system and are core areas involved in reward processing more broadly (Liu et al., 2011).

There is also converging evidence from event-related potential (ERP) research, particularly using the reward positivity [i.e., RewP; known previously as the feedback negativity (FN), or feedback-related negativity (FRN); Proudfit, 2015], as an index of reward dysfunction in depression. The RewP, which reflects the initial binary evaluation of outcomes as either better or worse than expected (Hajcak et al., 2007; Holroyd et al., 2008), is blunted in both clinical (Liu et al., 2014; Brush et al., 2018; Mulligan et al., 2018) and nonclinical samples (Bress et al., 2012; Mulligan et al., 2018), as well as among individuals with low self-reported reward sensitivity (Bress and Hajcak, 2013). Diminished RewP amplitude may also represent a neurobiological mechanism of risk for depression, such that it is more prevalent among people with a family history of depression (Foti et al., 2011; Kujawa et al., 2014) and has been shown to predict first episode onset of MDD (Bress et al., 2013; Nelson et al., 2016).

There are multiple reward stimuli types that can be leveraged for use in experimental research. For example, behavioral neuroscience studies typically use primary rewards or direct stimulation of reward-related regions to manipulate behavior (Salamone et al., 1994; Garris et al., 1999; Assadi et al., 2009). Yet, translational research of reward functioning in humans results in most studies conceptualizing reward narrowly, usually in terms of winning a nominal amount of money on laboratory tasks (i.e., monetary rewards; Liu et al., 2011). In fact, most reward processing studies in depression have used monetary contingencies to elicit reward-related behavior and neural activity (e.g., Knutson et al., 2008; Pizzagalli et al., 2008; Smoski et al., 2011; Ait Oumeziane and Foti, 2016). The emphasis on monetary rewards may in part be due to the relative ease of manipulating contingencies and eliciting neural responses. Nevertheless, findings based on a limited range of secondary rewards are then incorporated in general theories of reward dysfunction in depression. Monetary rewards are *assumed* to capture general reward functioning and studies have shown that primary (e.g., food) and secondary (e.g., money) rewards activate a common neural network (Sescousse et al., 2013). This focus on monetary rewards precludes a broader understanding of the role of social decision-making and reward functioning in depression (Forbes, 2009). Clarifying whether laboratory-based measures capture global or domain-specific reward deficits can have important implications for treatment. Global deficits may be indicative of efficient treatment targets with broad clinical utility (i.e., multiple psychopathologies, including substance use disorders, mood disorders, and schizophrenia), whereas deficits that are domain-specific may facilitate more targeted interventions based on the idiosyncratic profile of functional impairment at the individual level. A critical gap, however, is that studies of abnormal reward sensitivity in depression have largely assumed that laboratory-based based measures capture a global deficit, rather than directly comparing sensitivity to different reward types.

Far less is known about the regulation of neural responses to social stimuli than for other rewards (i.e., money), which is a key gap given the importance of social rewards in human functioning and their capacity to shape behavior (Fehr and Camerer, 2007; Gunaydin et al., 2014). However, there has been a growing focus in recent research to elucidate the neural correlates of social reward processing (Forbes and Dahl, 2012; Guyer et al., 2012; Bhanji and Delgado, 2014). Social rewards, such as stimuli indicating acceptance (Olino et al., 2015) and peer feedback (Guyer et al., 2012), elicit similar patterns of neural activity (e.g., striatum) as seen in studies examining money rewards. Other studies showed that receiving monetary rewards and another individual’s positive opinion of oneself recruited similar striatal activity within the same sample (Izuma et al., 2008). Parallel findings from recent ERP studies showed that social and nonsocial reward elicited morphologically similar ERPs (Ait Oumeziane et al., 2017; Ethridge et al., 2017; Distefano et al., 2018). Together, these studies suggest different classes of rewards are underlined by an overlapping neural system or “common neural currency.”

Recent work in the literature has also advanced the argument that social rewards may be particularly significant in depression (Forbes, 2009; Forbes and Dahl, 2012). Impairment in social functioning is a prominent feature of depression (Badcock and Allen, 2003) wherein individuals commonly display diminished motivation to engage in social interactions (Davey et al., 2008). Social contexts contribute to the development of depression. For example, a loss of an intimate partner is a common precipitating event for first episode onset (Monroe et al., 1999), whereas social factors in adolescence influence both the onset and course of depression (Sheeber et al., 2001; Davey et al., 2008). Although social withdrawal limits the likelihood of experiencing social rewards, it is also possible that reward responsiveness to social stimuli in depression is less sensitive thereby representing a potentially relevant sub-process for social functioning. To date, only a few studies have explicitly examined social reward deficits in depression. In one study, dysphoric individuals mobilized less effort when expecting social approval (Brinkmann et al., 2014). Using a Chatroom Interaction task, youth at higher risk for depression displayed decreased reward-related striatal activity when being accepted by peers (Olino et al., 2015). Early findings implementing both social and monetary reward in ERP research shows dysphoric symptomatology was associated with diminished in RewP amplitude following female social feedback; participants completed the reward task under the pretense of receiving actual peer feedback (Distefano et al., 2018). Collectively, findings suggest depression is linked to impaired social and nonsocial reward functioning. A key gap, however, is that no study to date has evaluated social and nonsocial reward sensitivity across a broad range of processing (i.e., reward anticipation and receipt) in depression within the same sample. Indeed, the present study seeks to extend past research by clarifying whether reward dysfunctions in depression are general (i.e., spanning both monetary and social reward) or domain-specific (i.e., stronger for social or monetary reward).

In addition to evaluating different reward types, there is also growing interest in characterizing reward-related reactivity across different phases of processing. Findings from basic neuroscience literature suggest that reward processing reflects a set of interrelated processes that unfold over time across multiple stages (Schultz, 2007), which are neurobiologically and functionally distinct (Berridge et al., 2009). Using an established reward paradigm [i.e., monetary incentive delay (MID)] originally developed for fMRI research (Knutson et al., 2000, 2001), past research leveraged the millisecond temporal resolution of ERPs to capture a broad range of reward-related neural responses (Novak and Foti, 2015). Notably, the MID task refined for ERP research disentangles distinct sub-stages *within* both anticipatory and consummatory reward processing, providing additional precision of reward dynamics over the traditional magnetic resonance imaging (MRI) version of the task.

The task structure within the incentive delay framework is ideal for systematically capturing a broad range of reward processing. First, a cue signals the contingency for that trial (incentive vs. neutral), followed by a target stimulus that requires a behavioral response (e.g., button press). On incentive trials, fast button presses yield a reward (e.g., monetary gain) whereas slow responses yield a non-reward (e.g., monetary loss). On neutral trials, participants break-even regardless of reaction time. Neural response to rewards during the MID can be indexed by multiple candidate ERP components. First, reward-predicting cues elicit an increased P3 (cue-P3) compared to neutral cues (Broyd et al., 2012; Novak and Foti, 2015). The P3 is maximal at parietal sites approximately 300–500 ms. The cue-P3 is thought to track the allocation of attentional resources toward reward-predicting cues. Following the cue-P3, a contingent negative variation (CNV) is elicited to reflect a shift from initial reward cue detection toward approach-motivated action preparation (Novak and Foti, 2015). The CNV is a sustained, negative-going ERP that is maximal at central electrodes in anticipation of a cued motor response (Rohrbaugh et al., 1976; Brunia et al., 2012) and is increased for reward vs. neutral trials (Novak and Foti, 2015). Monetary reward contingencies can also modulate the anticipation of feedback. A promising index is the stimulus preceding negativity (SPN), which is a sustained centroparietal negativity that is maximal prior to feedback onset (Ohgami et al., 2006; Brunia et al., 2012; Foti and Hajcak, 2012; Novak et al., 2016). Collectively, these ERPs tease apart reward anticipation into discriminable stages.

Consummatory reward processing, meanwhile, is indexed by two ERPs elicited by reward delivery. First, a RewP is apparent at the frontocentral electrodes and peaks 250–300 ms following feedback. Although initially thought to be a loss-related signal (i.e., FN/FRN; Miltner et al., 1997; Gehring and Willoughby, 2002) recent findings suggest that the RewP is modulated by reward outcomes: a positivity that is increased for rewards vs. non-rewards (Holroyd et al., 2008; Foti et al., 2011). Immediately following the RewP is the feedback-P3 (fb-P3). Like the cue-P3, the fb-P3 is maximal at parietal sites and peaks between 300 and 500 ms following stimulus onset; whereas the cue-P3 tracks the salience of reward-predicting cues, the fb-P3 tracks the salience of uncertain outcomes (i.e., it is increased for uncertain monetary gains and losses vs. certain “break-even” outcomes). On our task, RewP tracks outcome valence (win vs. loss) and fb-P3 tracks outcome uncertainty (win/loss vs. neutral; Novak and Foti, 2015).

In our own work, we adapted the MID tasks in Novak and Foti (2015) to examine peoples’ neural response to positive social feedback [i.e., Social Incentive Delay (SID); Ait Oumeziane et al., 2017]. Social rewards were defined positive performance feedback (i.e., “like”) in a social/interpersonal context; people completed the SID under the pretense that feedback was delivered in real-time by a peer so that they would seemingly value receiving positive and negative feedback from others. That is, the pretense of stimulated live feedback regarding participants’ performance was manipulated to be more evaluative than feedback generated automatically by a computer. This evaluative approach is in-line with a broader literature highlighting social-evaluative sensitivity in depression. For example, there is some evidence that depressed adults seek out excessive reassurance regarding their relationships and heavily rely on social approval for a sense of self-worth (Barnett and Gotlib, 1988; Joiner and Metalsky, 1995; Sheppard and Teasdale, 2004). Cognitive theories of depression have underlined the importance of sensitivity to feedback (e.g., social evaluation) as a potential vulnerability factor for depression (Beck, 1983; Mathews and MacLeod, 2005; Gotlib and Joormann, 2010). It is thought that depressed individuals may fail to utilize negative feedback to guide future performance (Elliott et al., 1997; Holmes and Pizzagalli, 2007; Steele et al., 2007), which could reflect underlying deficits in motivation (Eshel and Roiser, 2010). Other variants of the SID have utilized smiling faces as the feedback stimuli (Spreckelmeyer et al., 2009; Rademacher et al., 2010; Flores et al., 2015), which likely conflates reward and face processing. In addition, participants completing these tasks are cognizant of the notion that performance feedback was automated rather than determined by peers, thereby diminishing the social evaluative nature of the feedback. Here, we directly compare performance feedback in depression across multiple domains (social/nonsocial) and stage of processing.

Within this multi-faceted incentive delay framework, we demonstrated that social rewards on the SID elicited morphologically and psychometrically comparable ERPs as on the MID task in the same sample (Ait Oumeziane et al., 2017). Moreover, analogous ERPs across tasks (e.g., RewP on SID and MID) were moderately associated with one another (*r*’s 0.39–0.44), thereby highlighting the possibility of both a “common neural currency” and unique reward-type specific variance. That is, small correlations would suggest that these ERPs are primarily modulated by task-specific variability, whereas large correlations would indicate that there is little task-specific variability. The observed moderate correlations suggest the contribution of both general and task-specific reward sensitivity. Indeed, the combination of the SID and MID may have the potential of facilitating a more nuanced understanding of reward-related social and nonsocial neural dysfunctions in depression.

The current study seeks to systematically assess how depressive symptom severity relates to neural sensitivity to both social and monetary rewards within the same sample across a broad range of processing (reward anticipation and receipt). First, we aim to replicate our previous findings showing that ERPs elicited by social and monetary rewards on the SID and MID, respectively, are comparable across tasks (Ait Oumeziane et al., 2017). We expect that ERPs across tasks will exhibit a pattern of neural activity consistent with a common neural network (Izuma et al., 2008); that is, analogous ERPs on SID and MID will be morphologically similar and moderately correlated with one another (e.g., potentiated SPN on SID will be correlated with enhanced SPN on MID; Ait Oumeziane et al., 2017).

Next, we sought to evaluate the relationship between depressive symptoms with social and nonsocial reward-related brain activity. Although prior research suggests that depression is associated with deficits in both social (Olino et al., 2015; Distefano et al., 2018) and monetary rewards (Foti and Hajcak, 2012), no study has shown whether these deficits manifest within the same sample, particularly in the context of anticipatory (i.e., cue-P3, CNV, SPN) and consummatory ERPs (i.e., RewP, fb-P3). We expected that depression would exhibit deficits in abnormal consummatory (e.g., RewP; Brush et al., 2018; Mulligan et al., 2018) reward sensitivity. In order to distinguish the specificity of reward dysfunction across general depression severity as compared to the anhedonic features, we also tested whether blunted reward ERPs mapped on to diminished self-reported reward responsiveness (i.e., trait-like anhedonia). We expected that ERPs more uniquely map on to reward responsiveness rather than depression more generally (Pizzagalli et al., 2005; Foti et al., 2011; Bress and Hajcak, 2013).

Here, we formally tested whether reward-type (i.e., social, monetary) moderates the relationship with depressive symptoms and reward responsiveness, separately. Complementing these analyses, we sought to examine whether task-specific variability is uniquely associated with self-report symptoms. Task-specific effects were isolated using a series of exploratory regressions wherein analogous ERPs across tasks were entered as simultaneous predictors (i.e., social and nonsocial ERPs was controlled for in each regression model) of depressive symptoms and reward responsiveness.

## Materials and Methods

### Participants

Demographic information is presented in Table 1. Participants were 121 adult volunteers. Participants were excluded due to past-month psychotropic medication use (*N* = 11). On SID, participants were excluded due to equipment failure (*N* = 2) and poor-quality ERP data (e.g., slow waves; *N* = 1), leaving 107 participants for the final analyses. On MID, participants were excluded for not completing the task (*N* = 4), equipment failure (*N* = 1), and poor-quality ERP data (*N* = 1), leaving 104 in the final analyses. There were 102 participants (*M* age = 19 years, *SD* = 1.15). with complete social and monetary reward ERP data in the final sample. Notably, participants in the current study represent a non-overlapping sample relative to our initial study comparing SID and MID ERPs (Ait Oumeziane et al., 2017).

**Table 1:** Sample characteristics.

Variable	*N*	%
Gender		
Male	58	53.2
Female	51	46.8
Race		
Caucasian	81	74.3
Asian	21	19.3
African American	4	3.7
Native Hawaiian/Pacific Islander	1	0.9
Ethnicity		
Hispanic/Latino	11	10.1
Non-Hispanic/Latino	94	86.2

### Measures

#### Center for Epidemiological Studies—Depression Scale (CES-D; Radloff, 1977)

The Center for Epidemiological Studies-Depression Scale (CES-D) is a 20-item self-report questionnaire intended to measure current levels of depressive symptomatology in the general population (Radloff, 1977). Participants were asked to rate each question based on how frequently during the past week each item applied to them. Each item was scored on a 4-point Likert-type scale of 0 (*rarely or none of the time*) to 3 (*most or all of the time*). Higher scores on the scale denote greater depressive symptoms. In the current sample, Cronbach’s α was 0.91.

#### Reward Responsiveness Scale (RR; Van den Berg et al., 2010)

The RR is an 8-item questionnaire used to quantify trait tendencies to engage in reward-related behavior (Van den Berg et al., 2010). This scale was developed as a means of providing a pure and more reliable measure of reward responsiveness than other self-report scales. Participants evaluate items on a Likert-scale from 1 (*strong disagreement*) to 4 (*strong agreement*). Higher scores denote greater reward responsiveness traits. In the current sample, Cronbach’s α was 0.91.

### Laboratory Tasks

#### Social Incentive Delay

**Figure 1:**
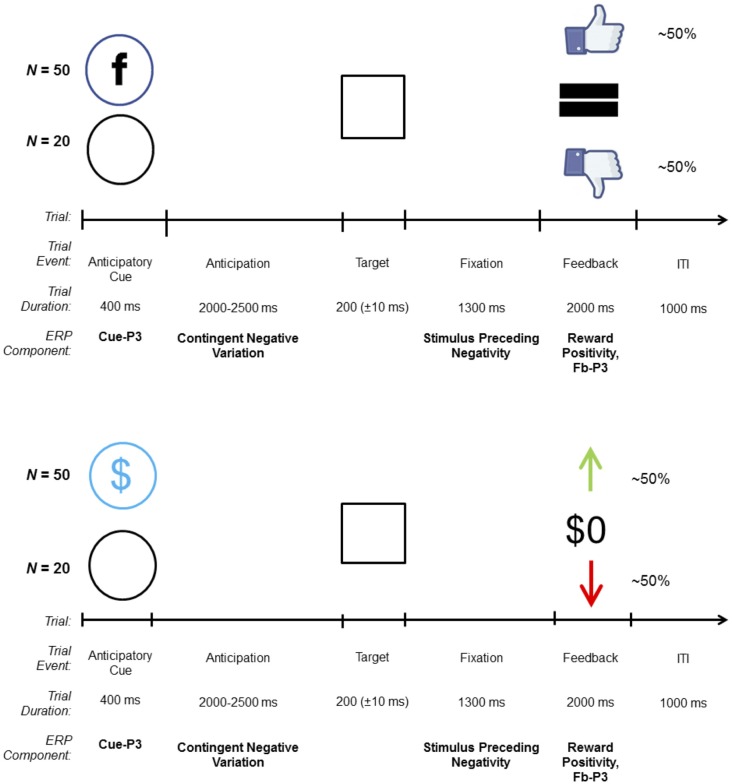
Trial structure and related event-related potential (ERP) components for the social [top; social incentive delay (SID)] and monetary [bottom; monetary incentive delay (MID)] incentive delay tasks. On each trial, one of two possible cues was presented: incentive (SID, MID) or neutral (empty circle). Target duration began at 200 ms and was dynamically adjusted based on task performance. On incentive trials, win and loss feedback were uncertain and based on performance; on neutral trials, feedback [i.e., “=” (SID), “$0” (MID)], was certain and predictable. The total number of trials depicted do not factor in the practice trials (*N* = 10) on SID and MID, and the overinclusion trials (*N* = 10; always receive positive social feedback to allay discomfort) on SID, all of which were not included in the analyses.

The SID task (Ait Oumeziane et al., 2017) was modeled after monetary reward tasks used in ERP research (Novak and Foti, 2015). An overview of the trial structure is shown in Figure 1. On each trial, participants were presented with one of two cues indicating the contingency for that trial: a blue circle with the letter “F,” similar to the Facebook logo, indicated a social contingency (i.e., possible positive or negative social evaluation; *N* = 50) and an empty circle indicated a neutral trial (i.e., no social evaluation; *N* = 20). Cues were followed by an anticipatory interval that varied in length from 2,000 to 2,500 ms, during which a fixation mark (“+”) was presented. The target stimulus (i.e., white box) was then presented; each participant was instructed to quickly click the left mouse button when the target appeared on the screen. After target offset, the fixation mark was presented for 1,300 ms while participants awaited feedback about their response. On incentive trials, successful responses resulted in a thumbs up (i.e., social media “like”) indicating a positive social evaluation, while unsuccessful responses resulted in a thumbs down (i.e., social media “dislike” or “unlike”) indicating a negative social evaluation. Neutral trials always resulted in no social evaluations “=.” Here, we used “thumbs up” and “thumbs down” as social feedback stimuli to perceptually mirror the “up” and “down” arrow used as the monetary feedback stimuli in the MID task, respectively. Although feedback was mirrored perceptually across tasks, ultimately different stimuli were selected in order to ensure that task differences were salient. It is possible that participants who complete the monetary reward task first inadvertently believe that positive feedback on SID yields monetary rewards. Feedback stimuli were presented for 2,000 ms, and the inter-trial interval was 1,000 ms. Task difficulty was adjusted to keep performance at approximately 50%; the target presentation became easier (+10 ms) following each unsuccessful response and more difficult (−10 ms) after each successful response.

Prior to starting the SID, participants were told that research assistants would use a computer program outside of the EEG booth to evaluate their performance on “social rounds.” To emphasize the role of the research assistants, participants were asked to treat the structure of the task similarly to how social media functions. For example, receiving a “like” by one’s peers on Facebook for sharing content (e.g., status update, photos) parallels how they will receive “thumbs up” feedback if the research assistant approved or “liked” their performance on “social rounds.” In reality, feedback stimuli were automated, and no real-time social evaluations were delivered. A practice block of 10 trials (eight incentives, two neutral) was used to determine initial task difficulty. Halfway through the task, participants received a short break. Ten consecutive incentive trials were added at the end of SID in order to allay any feelings of discomfort experienced from perceived negative social feedback; these trials were excluded from the analyses.

#### Monetary Incentive Delay

The overall trial structure, including the sequence and timing of all stimuli, was identical to the SID task; however, the cue and feedback stimuli differed (see Figure 1). On each trial, participants were presented with one of two cues indicating the contingency for that trial: a circle with a dollar symbol indicated a monetary incentive (i.e., possible gain or loss; *N* = 50 trials) and an empty circle indicated a neutral trial (i.e., certain break-even; *N* = 20 trials). On incentive trials, correct responses resulted in a green “↑” denoting a monetary gain of $0.40, while incorrect responses resulted in a red “↓” indicating a monetary loss of $0.20. Neutral trials always resulted in break-even feedback ($0). As before, a practice block of 10 trials (eight incentive, two neutral) was used to determine initial task difficulty. Halfway through the task, participants received a break; however, unlike SID they were informed of their cumulative winnings. Presentation software (Neurobehavioral Systems Inc., Berkeley, CA, USA) was used to control the timing and presentation of all stimuli for MID and SID.

### Procedure

After a short description of the experiment, EEG sensors were attached. Participants performed the reward tasks (i.e., SID, MID) and other tasks unrelated to this study, with task order counterbalanced across participants. After the experiment, participants completed the CES-D and RR measures and were paid their winnings (i.e., $5.00).

### Psychophysiological Recording and Data Reduction

The EEG was recorded *via* 32 Ag/AgCl active scalp electrodes using an actiCAP and the actiCHamp system (Brain Products, Munich, Germany). EEG signals were digitized at a 24-bit resolution with a sampling rate of 500 Hz. Impedances were maintained below 30 kOhm. Recordings were obtained from 32 scalp electrodes and a ground at Fpz. Vertical electrooculogram was recorded using two facial electrodes. Horizontal electrooculogram was recorded from electrodes FT9/10. Off-line analysis was performed using Brain Vision Analyzer software (Brain Products, Munich, Germany). All signals were re-referenced to the mastoid average (TP9/10) and band-pass filtered from 0.1 to 30 Hz. For the cue-P3 and CNV, the signal was segmented from −200 to 3,000 ms relative to cue onset. For the SPN, the signal was segmented from −1,700 to 100 ms relative to feedback onset (i.e., −200 to 1,600 relative to target onset). For the RewP and fb-P3, the signal was segmented from −200 to 1,000 ms relative to feedback onset. Each trial was corrected for blinks and eye movements (Gratton et al., 1983). Artifact rejection was conducted using a semi-automated procedure, with artifacts defined as: a step of 50 μV, >200 μV difference within 200-ms intervals, and <0.5 μV difference within 100-ms intervals. Additional artifacts were then identified using visual inspection.

ERPs were averaged separately for each condition on both tasks and corrected relative to their respective baseline windows (cue-P3 and CNV: −200 to 0 ms before cue onset; SPN: −1,200 to −1,000 ms before feedback onset; RewP and fb-P3: −200 to 0 ms before feedback onset). The average number of trials remaining for each condition after artifact rejection was as follows for SID: (1) social incentives for cue-P3 and CNV (*M* = 42.41 trials, *SD* = 4.45); (2) neutral incentive condition for cue-P3 and CNV (*M* = 16.56 trials, *SD* = 2.59); (3) social (*M* = 44.27 trials, *SD* = 4.80) and neutral (*M* = 17.23 trials, *SD* = 2.64) conditions for SPN; (4) positive (*M* = 21.50 trials, *SD* = 3.48) and negative (*M* = 20.88 trials, *SD* = 3.55) social outcomes for the RewP and fb-P3; and (5) neutral social outcome condition for fb-P3 (*M* = 17.10 trials, *SD* = 2.67). The average number of trials for MID was as follows: (1) monetary incentives for cue-P3 and CNV (*M* = 42.05 trials, *SD* = 5.46); (2) neutral incentive condition for cue-P3 and CNV (*M* = 16.76 trials, *SD* = 2.49); (3) monetary (*M* = 44.50 trials, *SD* = 4.50) and neutral (*M* = 17.84 trials, *SD* = 1.90) incentive conditions for SPN; (4) monetary gain (*M* = 22.82 trials, *SD* = 2.87) and loss (*M* = 20.75 trials, *SD* = 3.20) conditions for the RewP and fb-P3; and (5) neutral monetary outcomes for fb-P3 (*M* = 17.72 trials, *SD* = 1.99).

ERPs were scored using time-window averages, which was determined based on peak of the difference wave for each component within each task separately for the full sample. Given that we utilized distinct incentive cues and feedback stimuli across tasks, in addition to our findings from our development of the SID task (Ait Oumeziane et al., 2017), we expected that the time-window for the cue-P3, RewP, and fb-P3 may slightly differ across tasks. Thus, we scored each ERP surrounding the peak of relevant difference wave, regardless of their temporal properties of their counterpart component on the other task. Time windows and electrode poolings for MID ERPs were as follows: (1) cue-P3 from 390 to 440 ms after cue onset at Cz, CP1, CP2, Pz; (2) CNV from 2,200 to 2,400 ms after cue onset at FC1, Cz, C3, CP1; (3) SPN from −200 to 0 before feedback onset at Cz, CP1, CP2, Pz; (4) RewP from 250 to 300 ms post feedback at Fz, FC1, FC2, Cz; (5) fb-P3 from 340 to 490 ms post feedback at Cz, CP1, CP2, Pz. Time windows SID ERPs were as follows: (1) cue-P3 from 325 to 375 ms after cue onset; (2) the CNV from 2,200 to 2,400 ms after cue onset; (3) the SPN from −200 to 0 before feedback onset; (4) the RewP (i.e., positive minus negative outcome) from 290 to 340 ms post feedback; (5) the fb-P3 from 340 to 390 ms post feedback. The electrode poolings for SID ERPs were identical to MID.

### Data Analysis

Effects of condition and task on behavioral performance were evaluated using 2 (Task: MID vs. SID) × 2 (Condition) repeated-measured analysis of variances (ANOVAs). Effects of condition on ERP amplitudes were evaluated using 2 (Task: MID vs. SID) × 2 (Condition) × 2 (Task Order) repeated-measured ANOVAs. For anticipatory ERPs (cue-P3, CNV, and SPN), the effect of condition was tested by comparing incentive and neutral trials. For the RewP, the relevant condition contrast was the effect outcome valence (i.e., positive vs. negative outcomes). For the fb-P3, the relevant contrast was the effect of outcome salience (i.e., positive vs. neutral outcome, negative vs. neutral outcome). Follow-up contrasts to test for within task-modulation were performed for ERPs that showed a significant Task × Condition interaction.

As an alternative to subtraction-based ERP difference scores, we also used linear regression to create residualized neural responses to rewards controlling for non-reward conditions. For example, cue-P3_resid_ was created by saving the residual variance in a regression wherein cue-P3 on neutral trials was entered to predict cue-P3 on incentive trials. Other residualized anticipatory ERPs (i.e., CNV_resid_, SPN_resid_) followed the same steps (i.e., ERP on neutral trials predicting ERP on incentive trials). For RewP_resid_, RewP on loss trials was entered predicting the RewP on win trials. For fb-P3_resid_ to positive outcomes, fb-P3 on negative and neutral outcome trials were entered predicting fb-P3 on positive outcome trials. fb-P3_resid_ to negative outcomes, fb-P3 on positive and neutral outcome trials were entered predicting fb-P3 on negative outcome trials^1^. Each residual ERP difference score was calculated separately for SID and MID (e.g., RewP_resid_ on SID was calculated using only the relevant SID conditions).

To evaluate whether the association between depression symptoms and reward-related ERPs is moderated by reward type, we conducted a series of mixed-measure ANCOVAs. The within-subjects factor was Task (two levels; analogous SID and MID ERPs), whereas the between-subjects factor was self-report symptoms (i.e., CES-D and RR scores). In these models, the interaction between self-reported symptoms and task formally tests whether the strength of association differs across reward type. CES-D and RR scores were evaluated separated within each model. Next, a series of multiple linear regressions were performed to isolate task-specific variance in the instance of significant main effects of self-report symptoms and/or interaction between symptoms and task. These analyses complement the ANCOVAs, as regression is better suited to isolate unique task-specific variance in relation to depression. Within each regression model, analogous ERPs across tasks (e.g., RewP_resid_ on MID and SID) were entered as simultaneous predictors of depression or reward responsiveness scores. Each regression analysis also included effects task order, age, gender, and ethnicity as covariates.

## Results

### Sample Characteristics

Across the full sample, the average total CES-D score was 13.55 (*SD* = 10.01), with a range of 0–43. Approximately 108 (99%) participants in the sample reported at least some current symptoms (scores >0); 33 (30%) scored beyond the cut-off (>16) denoting higher risk for major depression. The average self-reported RR score was 26.83 (*SD* = 3.47), with a range of 18–32. RR and CES-D scores were not significantly correlated (*r* = −0.10, *p* = 0.15), likely due to the different time-frames of these scales.

#### Task Performance

The ANOVA revealed that reaction time varied as a function of Task (*F*_(1,104)_ = 15.30, *p* < 0.001, $\eta_{\text{p}}^{2}$ = 0.13). Overall, participants were quicker to respond on MID (*M* = 210.29 ms, *SE* = 2.83) compared to SID (*M* = 222.29 ms, *SE* = 3.56). There was also a significant main effect of Condition (incentive vs. neutral) across tasks (*F*_(1,104)_ = 106.66, *p* < 0.001, $\eta_{\text{p}}^{2}$ = 0.51). Participants were quicker to respond on incentive trials (*M* = 204.83 ms, *SE* = 2.57) compared to neutral trials (*M* = 227.75 ms, *SE* = 3.56). For SID, participants were significantly quicker on social (*M* = 211.95 ms, *SD* = 32.86) relative to neutral incentives (*M* = 232.02 ms, *SD* = 43.64; *t*_(106)_ = 7.78, *p* < 0.001, *d* = 0.75). For MID, reaction times were significant quicker on monetary (*M* = 197.68 ms, *SD* = 26.69) as compared to neutral incentives (*M* = 223.95 ms, *SD* = 37.85; *t*_(105)_ = 8.47, *p* < 0.001, *d* = 0.83). The Task × Condition interaction was not significant (*F*_(1,104)_ = 2.39, *p* = 0.13, $\eta_{\text{p}}^{2}$ = 0.02). As expected, participants were successful on 51.26% (*SD* = 3.00) and 50.39% (*SD* = 3.08) of all monetary and social incentive trials, respectively.

### Reward ERPs

#### Reward Anticipation

**Figure 2:**
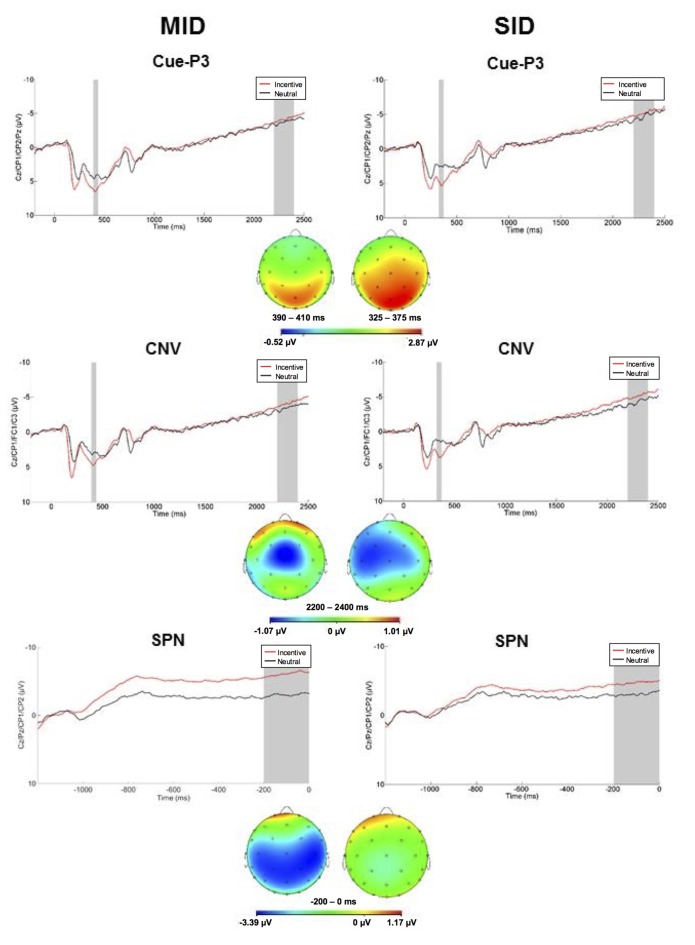
Left column: anticipatory ERP responses to monetary incentive and neutral trial conditions on MID. The cue-P3 was scored as the average activity in the first shaded window (top row: 390–440 ms) and the contingent negative variation (CNV) in the second shaded window (middle row; 2,200–2,400 ms). The stimulus preceding negativity (SPN; bottom row; −200 to 0 ms prior to feedback onset) was scored as the average in the shaded window. Right column: anticipatory ERP responses to social incentive and neutral trial conditions on SID. The cue-P3 was scored as the average activity in the first shaded window (top row: 325–375 ms) and the CNV in the second shaded window (middle row; 2,200–2,400 ms). The SPN (bottom row; −200 to 0 ms prior to feedback onset) was scored as the average in the shaded window. Below each waveform is the scalp distributions of the difference between incentive and neutral trials for the cue-P3 (top), CNV (middle), and SPN (bottom) for MID and SID.

Anticipatory ERPs are presented in Figure 2. Cues elicited a P3 that was maximal at centroparietal sites approximately 350 ms and 415 ms for SID and MID, respectively. The ANOVA yielded significant main effects of Task (*F*_(1,100)_ = 15.42, *p* < 0.001, $\eta_{\text{p}}^{2}$ = 0.13) and Condition (*F*_(1,100)_ = 72.74, *p* < 0.001, $\eta_{\text{p}}^{2}$ = 0.42); all other main effects, two-way, and three-way interactions were not significant (*p* > 0.10, $\eta_{\text{p}}^{2}$ < 0.05). Average cue-P3 amplitude (i.e., averaged across incentive and neutral conditions) was greater for MID (*M* = 5.36 μV, *SE* = 0.39) relative to SID (*M* = 3.79 μV, *SE* = 0.40). Furthermore, cue-P3 amplitude was more positive on incentive (*M* = 5.78 μV, *SE* = 0.37) compared to neutral cues (*M* = 3.37 μV, *SE* = 0.39).

Next, the CNV presented as a negative slow wave on MID and SID that was maximal immediately prior to target onset at left central electrodes. The CNV was sensitive to Condition (*F*_(1,100)_ = 4.33, *p* < 0.05, $\eta_{\text{p}}^{2}$ = 0.04); all other main effects, two-way, and three-way interactions were not significant (*p* > 0.10, $\eta_{\text{p}}^{2}$ < 0.10). The CNV was potentiated (i.e., more negative) on incentive (*M* = −4.68, *SE* = 0.49) compared to neutral trials (*M* = −3.92, *SE* = 0.50). Thus, CNV amplitude was modulated by incentive compared to neutral trials across tasks.

The SPN presented as a negative slow cortical wave immediately before feedback onset at the centroparietal sites. The ANOVA yielded a significant main effect of Condition (*F*_(1,100)_ = 48.74, *p* < 0.001, $\eta_{\text{p}}^{2}$ = 0.33) and Task × Condition interaction (*F*_(1,100)_ = 10.20, *p* < 0.01, $\eta_{\text{p}}^{2}$ = 0.09); all other main effects, two-way, and three-way interactions were not significant (*p* > 0.10, $\eta_{\text{p}}^{2}$ < 0.10). On MID, SPN amplitude was more negative on monetary incentive (*M* = −5.90 μV, *SD* = 5.73) compared to neutral trials (*M* = −3.09 μV, *SD* = 4.78), *t*_(101)_ = 7.28, *p* < 0.001, *d* = 0.74. Similarly, SPN amplitude was more negative on social incentive (*M* = −4.59 μV, *SD* = 5.42) relative to neutral trials (*M* = −3.17 μV, *SD* = 5.22) on SID, *t*_(101)_ = 4.01, *p* < 0.001, *d* = 0.40. SPN amplitude on incentive trials was more negative on MID compared to SID, *t*_(101)_ = 2.85, *p* < 0.01, *d* = 0.28. Thus, the SPN functioned similarly in anticipation of monetary and social reward outcomes, although reward-related anticipation was greater for monetary rewards.

#### Reward Receipt

**Figure 3:**
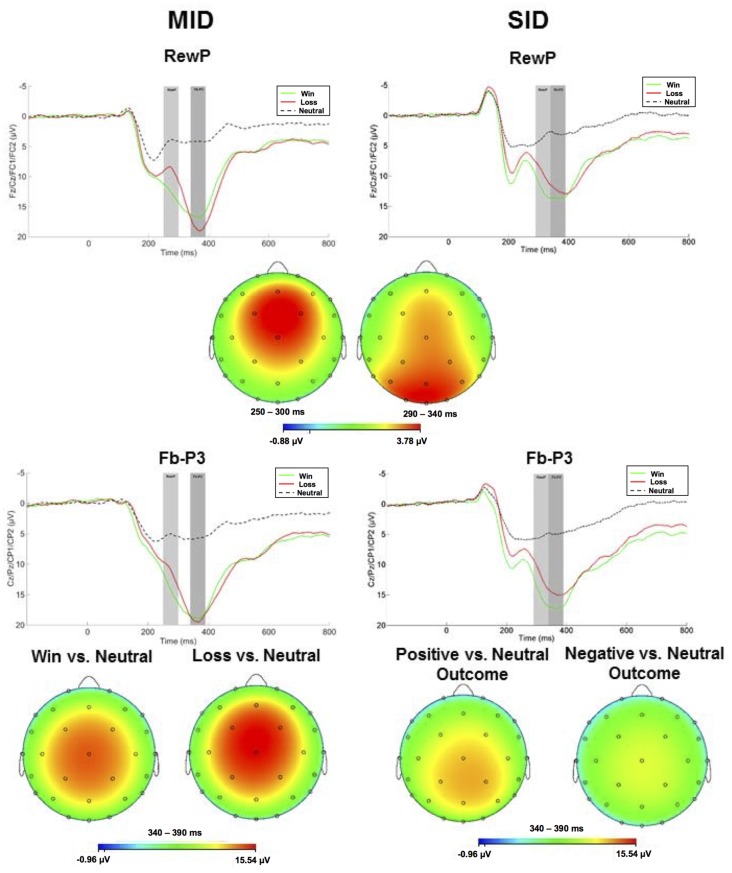
Left column: ERP responses to monetary wins, losses, and neutral outcomes on MID. Feedback onset was at 0 ms. The RewP was scored as the average activity in the first shaded window (top row: 250–300 ms) and the fb-P3 in the second shaded window (bottom row 340–390 ms). Right column: ERP responses to positive, negative, and no social feedback on SID. The RewP was scored as the average activity in the first shaded window (top row: 290–340 ms) and the fb-P3 in the second shaded window (bottom row: 340–390 ms). Scalp distributions of the difference between conditions for the RewP (positive minus negative outcome) and fb-P3 (positive minus neutral; negative minus neutral) are depicted below each waveform for MID and SID.

ERPs evoked by feedback delivery are presented in Figure 3. The RewP was maximal at frontocentral sites approximately 275 ms and 315 ms for MID and SID, respectively. RewP amplitude was sensitive to Condition (positive vs. negative outcome; (*F*_(1,100)_ = 108.41, *p* < 0.001, $\eta_{\text{p}}^{2}$ = 0.52); all other main effects, two-way, and three-way interactions were not significant (*p* > 0.05, $\eta_{\text{p}}^{2}$ < 0.05). Across MID and SID, RewP amplitude was more positive on win trials (*M* = 11.99 μV, *SE* = 0.60) than loss trials (*M* = 8.90 μV, *SE* = 0.60)^2^.

Following the RewP, the fb-P3 peaked at 365 ms with a centroparietal scalp distribution for MID and SID. Fb-P3 amplitude to positive and negative outcomes across MID and SID were analyzed as separate ANOVAs. For fb-P3 amplitude to positive outcomes (i.e., monetary and social), there was a significant main effect of Task (*F*_(1,100)_ = 10.87, *p* < 0.01, $\eta_{\text{p}}^{2}$ = 0.10) and Condition (*F*_(1,100)_ = 520.21, *p* < 0.001, $\eta_{\text{p}}^{2}$ = 0.84); all other main effects, two-way, and three-way interactions were not significant (*p* > 0.10, $\eta_{\text{p}}^{2}$ < 0.05). Fb-P3 amplitude to positive outcomes was greater on MID (*M* = 12.08 μV, *SE* = 0.45) compared to SID (*M* = 10.75 μV, *SE* = 0.45), whereas fb-P3 amplitude was greater on positive outcome conditions (*M* = 17.45 μV, *SE* = 0.38) relative to neutral conditions (*M* = 5.38 μV, *SE* = 0.38) across both tasks; all other main effects, two-way, and three-way interactions were not significant (*p* > 0.05, $\eta_{\text{p}}^{2}$ < 0.05).

Next, we were also interested in examining effects of fb-P3 amplitude to *negative* outcomes (i.e., monetary and social). There was a significant main effect of Task (*F*_(1,100)_ = 35.67, *p* < 0.001, $\eta_{\text{p}}^{2}$ = 0.26), Condition (*F*_(1,100)_ = 369.02 *p* < 0.001, $\eta_{\text{p}}^{2}$ = 0.79), and Task × Condition interaction (*F*_(1,100)_ = 32.57, *p* < 0.001, $\eta_{\text{p}}^{2}$ = 0.25); all other main effects, two-way, and three-way interactions were not significant (*p* > 0.10, $\eta_{\text{p}}^{2}$ < 0.05). Fb-P3 amplitude (i.e., ERP activity across negative and neutral outcome trials) was greater on MID (*M* = 12.33 μV, *SE* = 0.51) compared to SID (*M* = 9.59 μV, *SE* = 0.49), whereas fb-P3 amplitude was greater on negative outcome conditions (*M* = 16.54 μV, *SE* = 0.65) relative to neutral conditions (*M* = 5.38 μV, *SE* = 0.38) across both tasks. Unlike fb-P3 to positive outcomes, we performed follow-up contrasts for the significant Task × Condition interaction for the fb-P3 to negative outcomes. Results from *t*-test indicates that fb-P3 amplitude to monetary loss on MID (*M* = 18.89 μV, *SD* = 7.67) was significantly larger as compared to fb-P3 to negative social outcomes on SID (*M* = 14.14 μV, *SD* = 6.85), *t*_(101)_ = 7.46, *p* < 0.001, *d* = 0.74.

#### Links Between Social and Nonsocial Rewards

First, bivariate correlations were calculated between analogous residualized ERPs across tasks (e.g., RewP_Resid_ on MID with RewP_Resid_ on SID; see Table 2). The results indicated that residualized cue-P3, SPN, RewP and fb-P3 (i.e., positive and negative outcomes) amplitudes were significantly positively correlated across tasks. The cross-task correlation of CNV amplitude, however, was not significant.

**Table 2:** Correlations of analogous social and nonsocial event-related potentials (ERPs).

	*r*
1. Cue-P3_resid_	0.23*
2. CNV_resid_	0.11
3. SPN_resid_	0.41**
4. RewP_resid_	0.28**
5. Fb-P3 positive outcome_resid_	0.40***
6. Fb-P3 Negative outcome_resid_	0.37***

Note: correlations were calculated using residual ERP difference scores. The correlation coefficient (*r)* denote the relationship of analogous ERP components across social incentive delay (SID) and monetary incentive delay (MID). *p < 0.05, **p < 0.01, ***p < 0.001.

### Reward Processing and Internalizing Symptoms

#### ANCOVAs

Results across the ANCOVAs conducted are presented in Tables s 3, 4. Within each model, analogous SID and MID were entered as the within-subjects factor and self-report measures (CES-D, RR) were entered as between-subjects factor. Separate models were calculated for CES-D and RR scores for each ERP. First, there was a significant main effect of CES-D score when SPN (*F*_(1,100)_ = 5.29, *p* < 0.05, $\eta_{\text{p}}^{2}$ = 0.05) and fb-P3 amplitude to positive outcomes (*F*_(1,100)_ = 4.56, *p* < 0.05, $\eta_{\text{p}}^{2}$ = 0.05) were entered in the model. This indicates that the associations with CES-D were statistically similar across MID and SID tasks for the SPN and the fb-P3 to positive outcomes. Main effects and interactions with Task were not statistically significant^3^. Next, there was a significant main effect of RR score (*F*_(1,98)_ = 4.74, *p* < 0.05, $\eta_{\text{p}}^{2}$ = 0.05) when RewP amplitude was entered in the model, indicating a statistically similar association between RR and RewP across MID and SID tasks; all other main effects and interactions were not statistically significant^4^.

**Table 3:** Summary of ANCOVA analysis for anticipatory reward ERPs.

	Cue P3			CNV			SPN		
	Depression								
	*F*_(1,100)_	*p*	$\eta_{\text{p}}^{2}$	*F*_(1,100)_	*p*	$\eta_{\text{p}}^{2}$	*F*_(1,100)_	*p*	$\eta_{\text{p}}^{2}$
Task	0.04	0.85	0.00	0.19	0.66	0.00	0.61	0.44	0.01
CES-D	0.95	0.33	0.01	0.15	0.70	0.00	5.29*	0.02	0.05
Task CES-D	0.04	0.84	0.00	0.36	0.55	0.00	0.70	0.41	0.01∣rule
	**Reward responsiveness**								
	*F*_(1,98)_	*p*	$\eta_{\text{p}}^{2}$	*F*_(1,98)_	*p*	$\eta_{\text{p}}^{2}$	*F*_(1,98)_	*p*	$\eta_{\text{p}}^{2}$∣rule
Task	1.66	0.20	0.02	0.08	0.78	0.00	0.52	0.47	0.01
RR	0.06	0.81	0.00	1.47	0.23	0.02	0.33	0.56	0.00
Task RR	1.73	0.19	0.02	0.07	0.79	0.00	0.60	0.44	0.01

Note: CES-D denotes Center for Epidemiologic Studies Depression Scale. RR denotes reward. *p < 0.05.

**Table 4:** Summary of ANCOVA analysis for consummatory reward ERPs.

	RewP			Fb-P3 positive outcome			Fb-P3 negative outcome		
	Depression								
	*F*_(1,100)_	*p*	$\eta_{\text{p}}^{2}$	*F*_(1,100)_	*p*	$\eta_{\text{p}}^{2}$	*F*_(1,100)_	*p*	$\eta_{\text{p}}^{2}$
Task	0.00	0.96	0.00	0.31	0.58	0.00	0.01	0.94	0.00
CES-D	1.51	0.22	0.02	4.56*	0.04	0.04	3.03	0.09	0.03
Task × CES-D	0.09	0.76	0.00	0.63	0.43	0.01	0.03	0.85	0.00∣rule
	**Reward responsiveness**								
	*F*_(1,98)_	*p*	$\eta_{\text{p}}^{2}$	*F*_(1,98)_	*p*	$\eta_{\text{p}}^{2}$	*F*_(1,98)_	*p*	$\eta_{\text{p}}^{2}$∣rule
Task	0.02	0.90	0.00	0.78	0.38	0.01	0.34	0.56	0.00
RR	4.74*	0.03	0.05	3.00	0.09	0.03	0.22	0.22	0.02
Task × RR	0.03	0.87	0.00	0.80	0.37	0.01	0.35	0.56	0.00

Note: CES-D denotes Center for Epidemiologic Studies Depression Scale. RR denotes reward. *p < 0.05.

#### Regressions

Complementing these ANCOVAs, a series of multiple linear regressions were conducted to assess unique task-specific variability in depression (Table 5) and reward responsiveness (Table 6). Regressions analyses were performed only in instances where at least one main effect or interaction was significant in the ANCOVAs. Standardized analogous residualized ERPs across SID and MID were included as simultaneous predictors of CES-D and RR scores. Each regression model also contained the main effects of task order, age, gender, and ethnicity as covariates. In predicting CES-D scores, there was a significant main effect of fb-P3_resid_ to positive outcomes on SID but not MID. Specifically, blunted fb-P3_resid_ to positive social outcomes uniquely predicted higher depressive symptoms, over and above fb-P3 to monetary rewards. There were no significant effects for SPN amplitude on SID or MID. All covariate main effects were not statistically significant. Next, there was no significant effect of RewP_resid_ amplitude on SID or MID in predicting RR scores.

**Table 5:** Predicting unique reward-related neural deficits in depression.

	Outcome: depression score	
	Model 1: SPN_resid_	Model 2: Fb-P3_resid_
**Covariates**		
Task order	−0.04	−0.06
Age	−0.11	−0.07
Gender	−0.06	−0.04
Ethnicity	0.14	0.13
**SID ERPs**		
SPN_resid_	0.20	-
Fb-P3_resid_ positive	-	−0.25*
Social Outcomes
**MID ERPs**		
SPN_resid_	0.06	-
Fb-P3_resid_ monetary gains	-	0.00

Note: columns represent separate regression models wherein analogous ERPs across MID and SID were entered as simultaneous predicts of depression. Gender was coded as 0 = Male, 1 = Female. Ethnicity was coded as 0 = Hispanic, 1 = Other. Regression coefficients are denoted using standardized beta weights. SPN amplitude reflects a slow, sustained negativity; therefore, a positive beta weight with CES-D denotes the reverse association. *p < 0.05.

**Table 6:** Predicting unique reward-related neural deficits in self-report reward responsiveness.

	Outcome: reward responsiveness
	RewP_resid_
**Covariates**	
Task order	−0.04
Age	−0.09
Gender	0.15
Ethnicity	−0.11
**SID ERPs**	
RewP_resid_	0.13
**MID ERPs**	
RewP_resid_	0.14

*Note: gender was coded as 0 = Male, 1 = Female. Ethnicity was coded as 0 = Hispanic, 1 = Other. Regression coefficients are denoted using standardized beta weights*.

## Discussion

The current study is the first to systematically examine social and nonsocial reward-related neural dysfunction in depression within the same sample. We successfully replicated our previous efforts to elicit parallel reward-related neural activity to social and monetary rewards. The SID and MID tasks elicited morphologically similar ERPs across different stages of reward processing (i.e., reward anticipation, reward receipt) and were moderately associated with one another. We also extended the literature by leveraging the social and nonsocial reward ERP framework to the study of individual differences in depressive symptomatology and self-reported trait reward sensitivity. We demonstrated that depressive symptomatology was characterized by broad reductions in anticipation of uncertain outcomes (i.e., reduced SPN across SID and MID) and in the salience of positive outcomes (i.e., fb-P3 to monetary gains and positive social feedback), across reward types. We also showed that blunted consummatory social reward processing in the time-window spanning the RewP and fb-P3 amplitudes (i.e., positive social outcomes) was associated with reward responsiveness. Complementing these findings, there was also evidence of a task-specific association between depressive symptoms and the fb-P3 to positive social outcomes, controlling for monetary outcomes. Overall, the current study provides early evidence of both general and domain-specific (social) reward deficits in depression.

Here, we replicated previous efforts to utilize the incentive delay framework for social (Ait Oumeziane et al., 2017) and nonsocial ERP research on reward processing (Novak and Foti, 2015). Consistent with previous studies, we found that anticipatory (cue-P3, SPN) and consummatory ERPs (RewP, fb-P3) were modulated by incentive and reward outcomes, respectively, regardless of reward type. Analogous reward ERPs across SID and MID were also morphologically similar and moderately associated, highlighting the possibility of a “common neural currency.” Indeed, this finding is in concert with past fMRI (Izuma et al., 2008; Guyer et al., 2012) and ERP research (Ait Oumeziane et al., 2017; Ethridge et al., 2017; Distefano et al., 2018) that have suggested the social and monetary reward tap into an overlapping neural network.

These findings, however, are in light of evidence showing that ERP temporal onset was distinct across multiple stages of processing, including reward cue detection [i.e., cue-P3 (50 ms)] and initial evaluation of outcome valence [i.e., RewP (40 ms)]. Differences in stimuli properties may have contributed to these differences, as prior research has shown that stimulus complexity can impact the temporal properties of ERPs (Baker and Holroyd, 2011). Within each task, different ERP components were scored in non-overlapping time intervals; however, if stimuli properties impacted temporal onset, particularly in regard to RewP and fb-P3, then it is possible that the intervals scored may reflect a combination of distinct processes. Implementing distinct incentive and feedback stimuli was an important manipulation, in conjunction with participants completing the task under the pretense of live simulated peer feedback (Ait Oumeziane et al., 2017), insofar as to increase the social engagement and increase the value of receiving positive and negative feedback from others. It would be of interest for future research to explore the possibility of increasing the similarity in perceptual properties across SID and MID, although this may lead to other confounds. For example, it is possible that participants who complete the MID first may believe that SID feedback yield monetary rewards if identical stimuli are used across tasks.

The current study highlights that depression may be associated broadly with anticipatory and consummatory processing across social and nonsocial rewards. Specifically, depressive symptoms were linked to both reduced anticipation of uncertain outcomes (SPN across MID and SID) and blunted salience of positive feedback (fb-P3 to positive social feedback and monetary gains). The moderating effect of reward type was not significant, suggesting a generalizable effect across tasks. These parallel findings for the SPN and fb-P3 are consistent with past findings demonstrated that these two ERP components are intertwined, such that greater feedback anticipation predicts higher feedback salience (Novak et al., 2016). However, we extend the literature by highlighting that depression is broadly implicated by neural deficits to reward (social and monetary). Interestingly, unlike previous studies we did not find significant associations between depression and RewP amplitude, both in regards to general and domain-specific deficits. Past studies have shown that an attenuated RewP amplitude is associated with depression (Liu et al., 2014; Umemoto and Holroyd, 2017; Brush et al., 2018). The relationship between RewP amplitude is less direct and more nuanced than previously considered. For example, blunted RewP amplitude and depression may operate through other clinically related dimensions (Ait Oumeziane and Foti, 2016; Nelson et al., 2016; Novak et al., 2016). Alternatively, diminished RewP amplitude may be associated with a trait-like depression vulnerability rather than current symptom severity (Bowyer et al., 2019). In contrast to depression, our findings showed that reward lower reward responsiveness was associated with reduced RewP amplitude across social and monetary rewards. These differences may be due to the way positive affect is conceptualized; that is, RewP may be more sensitive to trait (RR) rather than state levels of positive affect (CES-D).

These findings provide preliminary evidence of general patterns of reward reactivity (i.e., both social and nonsocial) reward reactivity in depression. To further contextualize these results, we performed a series of multiple linear regressions to isolate task-specific MID and SID variance in relation to self-report symptoms. Our findings suggest that blunted salience to positive social feedback uniquely predicted depressive symptoms, over and above one’s fb-P3 amplitude to nonsocial rewards. Whereas blunted fb-P3 in our sample appears to be sensitive to social contexts, there was no significant effect from isolating task-specific variance for anticipation of outcomes (i.e., SPN) in predicting depression. Consistent with the ANVOCAs findings, depression may be characterized by general deficits in anticipation of uncertain outcomes (i.e., both social and nonsocial). It would be of interest for future research to evaluate this possibility of a latent reward dimension using advanced statistical technique such as structural equation modeling.

There is growing interest in assessing the role of social reward dysfunction in depression (Forbes, 2009; Forbes and Dahl, 2012). Past studies have demonstrated that depressed individuals exhibit blunted neural activation to social rewards (Olino et al., 2015); however, we addressed a key gap by showing that symptoms of depression may be uniquely related to diminished salience of positive social feedback, over and above other reward types. Interestingly, we did not observe any significant effect of monetary reward-related neural activity, which is in contrast with a multitude of studies implicating monetary reward processing deficits in depression (e.g., Liu et al., 2014; Umemoto and Holroyd, 2017; Brush et al., 2018). One possible explanation is that the SID task impacted the interrelationship between monetary reward sensitivity and depression in some manner; however, there was no significant effect of task order across our analyses. Alternatively, many ERP studies in depression have used simple guessing tasks (e.g., Foti and Hajcak, 2012; Ait Oumeziane and Foti, 2016), whereas the current study utilizes an active, performance-based task. It would be of interest for future studies to evaluate whether active vs. passive task properties within the same sample mediates the relationship between monetary ERPs and depression.

Nevertheless, gaining a more nuanced understanding of the neural correlates of reward processing in depression, beyond monetary contingencies, can have important treatment considerations. For example, recent work describes interventions [e.g., Positive Affect Treatment (PAT; Craske et al., 2016)] specifically designed to target deficits in reward sensitivity. Within this framework, blunted fb-P3 to positive social feedback may represent a novel target for treatment wherein attention is guided towards important in-the-moment factors (physical sensations, thoughts, behaviors, mood) during social contexts to facilitate increased engagement with reward. This notion, however, is speculative in nature as more research linking the therapeutic benefits on neural measures is required. Nevertheless, it does highlight the potential clinical utility of gaining a better understanding of the pathophysiological processes of depression, as doing so may shed light on more effective and targeted treatments (Forbes, 2009).

A strength of the current study is the use of theoretically distinct reward paradigms within a large sample (*N* = 107). The strengths should be considered in light of the limitations. First, the current study did not assess whether participants believed that peers were evaluating them in real-time; however, existing studies show imagined social feedback is sufficient in eliciting striatal activity (Hsu et al., 2013). Second, although we found evidence of task-specific (i.e., social) and general reward-related abnormalities in depression, it is that it is unclear how these effects extend to more severe populations. Nevertheless, subclinical depressive symptomology is highly prevalent (Cuijpers et al., 2004) and represents a significant risk factor for the onset of a major depressive episode (Cuijpers et al., 2004). These findings enhance our understanding of reward-related dysfunction in mood disorders by extending dimensional approaches of classification to subthreshold and healthy populations (Insel et al., 2010; Cuthbert and Insel, 2013). A second limitation is that the incentive delay framework is effective for capturing anticipatory and consummatory neural activity, but it cannot isolate other relevant reward processing, particularly reward learning. Previous research has linked depression with an impaired capacity to acquire reward contingencies (Pizzagalli et al., 2008; Herzallah et al., 2013; Vrieze et al., 2013). It would be of interest to apply the present framework in conjunction with existing reward learning paradigms to improve understanding of the full range of reward processing. Indeed, a more fine-grain understanding of reward dysfunction may help lay the foundation for identifying meaningful subgroups in depression characterized by disruptions in reward type (social, nonsocial), phase (reward anticipation, receipt, learning), or a combination of these factors.

Disruptions in reward-related functioning may play an important role in the pathophysiology of depression. The current study extends the literature by examining whether reward dysfunction in depression is general and/or domain-specific using theoretically distinct paradigms of social and nonsocial rewards. We demonstrated found that depression was characterized by deficits across two stages of processing: blunted anticipation of unexpected outcomes and salience of positive feedback. When simultaneously accounting for analogous neural activity, only blunted salience of positive social feedback was a significant predictor of depressive symptoms. Blunted anticipation to unexpected outcomes appeared to reflect general rather than task-specific reward variance. Overall, social reward sensitivity appears to be an important neural correlate that may enhance our understanding of the pathophysiology of depression. This study underscores the importance of a multi-faceted assessment of reward functioning toward the goal of understanding psychopathology, particularly in the context of depression.

## Data Availability

The datasets generated for this study are available on request to the corresponding author.

## Ethics Statement

This study was approved by Purdue University’s Human Research Protection Program. All participants gave full study consent prior to any research procedures.

## Author Contributions

BA and DF contributed to the conception and design of the study. BA collected the data, organized the database and performed the statistical analyses. BA, OJ, and DF contributed to writing and revision of the manuscript. All authors read and approved the submitted version.

## Conflict of Interest Statement

The authors declare that the research was conducted in the absence of any commercial or financial relationships that could be construed as a potential conflict of interest.

## Footnotes

^1^Our analyses showed a significant Task × Condition interaction for the fb-P3, thus we calculated the residual difference score different than when using subtraction methods. This difference was important insofar as to disentangle reward magnitude (fb-P3) and potential overlapping valence effects typically associated with the RewP.

^2^We performed follow-up test to determine whether the effect of Condition for RewP amplitude remained significant when covarying for fb-P3 amplitude to positive and negative social feedback. This analysis was important insofar as to control for potential overlap between RewP and fb-P3 components in the waveform. The results revealed a significant effect of Condition (Win vs. Loss: *F*_(1,99)_ = 5.09 *p* < 0.05, $\eta_{\text{p}}^{2}$ = 0.05), adjusting for fb-P3 on SID.

^3^We also conducted identical analyses by dichotomizing our sample into “healthy controls” (*N* = 76) and “individuals at higher risk for depression” (i.e., CES-D scores greater than 16; *N* = 33). We found a significant main effect of CES-D when SPN was entered for healthy controls (*F*_(1,68)_ = 7.96, *p* < 0.01, $\eta_{\text{p}}^{2}$ = 0.11) but not individuals at risk for depression (*F*_(1,30)_ = 0.57, p = 0.46, $\eta_{\text{p}}^{2}$ = 0.02). All other main effects and interactions were not statistically significant (i.e., *p* > 0.05).

^4^We also evaluated identical ANCOVAs for RR across “healthy controls” and “individuals at risk for depression.” Results showed no significant main effects or interactions for either group (i.e., *p*’s > 0.05).
